# Optimized Protocol for the Detection of Multifunctional Epitope-Specific CD4^+^ T Cells Combining MHC-II Tetramer and Intracellular Cytokine Staining Technologies

**DOI:** 10.3389/fimmu.2019.02304

**Published:** 2019-10-09

**Authors:** Gabiria Pastore, Monica Carraro, Elena Pettini, Emanuele Nolfi, Donata Medaglini, Annalisa Ciabattini

**Affiliations:** Laboratory of Molecular Microbiology and Biotechnology, Department of Medical Biotechnologies, University of Siena, Siena, Italy

**Keywords:** MHC-II tetramers, ICS, cytokines, multifunctional T cells, flow cytometry, immune response, vaccination

## Abstract

Analysis of multifunctional CD4^+^ T cells is fundamental for characterizing the immune responses to vaccination or infection. Major histocompatibility complex (MHC)/peptide tetramers represent a powerful technology for the detection of antigen-specific T cells by specific binding to their T-cell receptor, and their combination with functional assays is fundamental for characterizing the antigen-specific immune response. Here we optimized a protocol for the detection of multiple intracellular cytokines within epitope-specific CD4^+^ T cells identified by the MHC class II tetramer technology. The optimal procedure for assessing the functional activity of tetramer-binding CD4^+^ T cells was based on the simultaneous intracellular staining with both MHC tetramers and cytokine-specific antibodies upon *in vitro* restimulation of cells with the vaccine antigen. The protocol was selected among procedures that differently combine the steps of cellular restimulation and tetramer staining with intracellular cytokine labeling. This method can be applied to better understand the complex functional profile of CD4^+^ T-cell responses upon vaccination or infection.

## Introduction

The study of the CD4^+^ T-cell activation and effector function is fundamental in the characterization of immune responses to vaccination (1).

CD4^+^ T cells play a central role in mediating vaccine immune responses by shaping both the humoral and cellular immunity (2). Activated CD4^+^ T cells are critically involved in providing cognate help to B cells for the production of protective antibodies and modulate the functions of macrophages and CD8^+^ cytotoxic T cells through cytokines secretion. The characterization of the cytokine production of antigen-specific T cells is therefore of critical importance to profile vaccine immune responses. The direct and specific method for identifying antigen-specific CD4^+^ T cells is based on the major histocompatibility complex (MHC) tetramer staining technique (3). This procedure allows the identification of specific T cells due to the selective and multivalent binding of tetramer MHC–peptide complexes to the T-cell receptors (TCRs) (3, 4) and has been used for characterizing the primary and recall antigen-specific CD4^+^ T-cell responses in many pre-clinical and human studies (1, 5–9).

The effector function of antigen-reactivated T cells is commonly measured by flow cytometry-based intracellular cytokine staining (ICS) that allows simultaneous phenotypic characterization and cytokine detection within single cells (10, 11). The characterization of intracellular cytokines allows the identification of activated CD4^+^ T cells capable of producing more than one cytokine, and the analysis of these multifunctional/polyfunctional cells is important for characterizing the immune response elicited by vaccination or natural infection (12). Polyfunctional CD4^+^ T cells secreting interferon (IFN)-γ, tumor necrosis factor (TNF)-α, and interleukin (IL)-2 have been proposed as a major component of the immune response that correlates with mouse protection against challenge with *Leishmania major* (13). In tuberculosis (TB), it has not been elucidated if the frequency and quality of polyfunctional CD4^+^ T-cell responses elicited in mice by different types of vaccines correlate with protective immunity (14–17), while human studies have shown that a consistent response of CD4^+^ T cells coexpressing IFN-γ, TNF-α, and IL-2 was associated with acute TB infection (18). Tetramer labeling and intracellular cytokine staining are generally not recommended to be performed concurrently since the *in vitro* antigen restimulation can induce TCR internalization, thus losing the possibility of detecting epitope-specific CD4^+^ T cells using tetramers (19).

To identify a protocol for the detection of intracellular cytokine production within the activated epitope-specific CD4^+^ T cells, we assessed different strategies that combined cellular restimulation (with the vaccine antigen or tetramers) and tetramer staining (extracellular or intracellular) with intracellular cytokine labeling. The different procedures were tested in two different models. In the first one mice were immunized with the chimeric TB vaccine antigen H56 (20) mixed with the adjuvant CAF01 (21), a model vaccine formulation deeply characterized in pre-clinical studies for its capacity of inducing both humoral and cellular responses (9, 22–24). H56 is a fusion protein of *Mycobacterium tuberculosis* antigens Ag85B, ESAT-6, and Rv2660, and the H56-specific CD4^+^ T-cell response can be monitored by employing Ag85B_280−294_-complexed MHC class II tetramers (8). In the second experimental setting, mice were immunized with the model chicken ovalbumin antigen, and the CD4^+^ T-cell response was assessed employing tetramers specific for the epitope_325−335_ (25).

The comparative analysis of the different protocols has permitted to optimize the procedure for identifying the multifunctional profile of tetramer-specific CD4^+^ T cells by performing intracellular staining with both tetramers and cytokine-specific antibodies upon antigen restimulation. This method represents a helpful tool for identifying epitope-specific CD4^+^ T cells and analyzing their specific effector function.

## Materials and Methods

### Mice

Female C57BL/6 mice, purchased from Charles River (Lecco, Italy), were housed under specific pathogen-free conditions in the animal facility of the Laboratory of Molecular Microbiology and Biotechnology (LA.M.M.B.), Department of Medical Biotechnologies at University of Siena, and treated according to national guidelines (Decreto Legislativo 26/2014). The protocol was approved by the Italian Ministry of Health (authorization no. 1004/2015-PR, 22 September 2015).

### Immunizations

Groups of 10–12 mice were immunized by the subcutaneous route at the base of the tail with the chimeric TB vaccine antigen H56 (2 μg/mouse) combined with the adjuvant CAF01 (250 μg dimethyldioctadecylammonium and 50 μg trehalose dibehenate/mouse), and boosted with a lower dose of H56 alone (0.5 μg/mouse) 4 weeks later. H56 and CAF01 were kindly provided by Statens Serum Institut, Denmark. Another group of 6 mice immunized with albumin from hen egg white (OVA, 25 μg/mouse, Sigma-Aldrich), combined with the adjuvant CAF01, and boosted with OVA alone. The priming formulations containing antigens and CAF01 were injected in a volume of 150 μl/mouse of Tris 10 mM, while the boosting formulations containing H56 and OVA alone in a volume of 100 μl/mouse of 1X Dulbecco's phosphate buffered saline (1X PBS). Mice were sacrificed 5 days after boosting.

### Sample Collection and Cell Preparation

Spleens collected from mice were mashed onto 70 μm nylon screens (Sefar Italia, Italy) and washed in complete RPMI (cRPMI) medium [RPMI (Lonza, Belgium), 100 U/ml penicillin/streptomycin, and 10% fetal bovine serum (Gibco, USA)] for 10 min at 300 g at 4°C. Splenocytes were treated with red blood cell lysis buffer (1X, eBioscience, USA) for 4 min. Following centrifugation at 300 × g at 4°C for 10 min, cells were washed with 1X PBS and counted with a cell counter (Bio-Rad, USA).

### Protocols and Reagents

**Figure 1:**
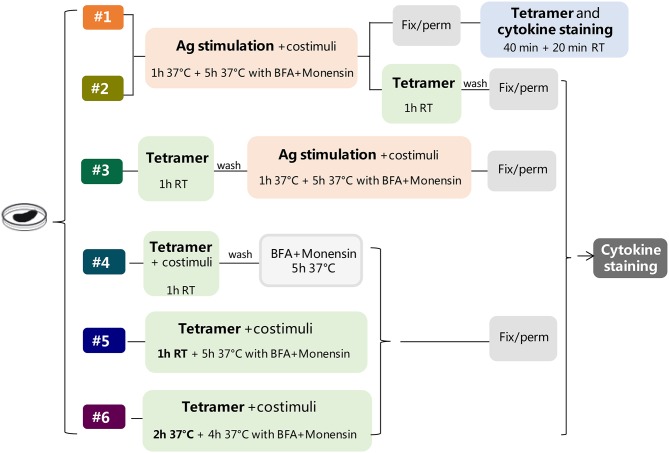
Study design. Six different protocols, combining antigen stimulation and tetramer staining with intracellular cytokine labeling, were used for detecting antigen-specific CD4^+^ T cells producing cytokines in splenocytes of mice immunized with two different antigens, H56 or OVA, and CAF01 adjuvant, 5 days after booster immunization. In protocols 1–3, splenocytes were restimulated with the respective antigen (Ag, pink box), added before (protocols 1 and 2), or after (protocol 3) tetramer staining (green box), while in protocols 4–6, the restimulation step was performed directly with Ag85B or OVA epitope-complexed MHC II tetramers. Anti-CD28 and anti-CD49d (costimuli) were added with Ag (protocols 1–3) or with tetramers (protocols 4–6). After Ag or tetramer incubation, cells were treated with brefeldin A and monensin for 4–5 h at 37°C, fixated and permeabilized (gray box), and finally stained with anti-cytokine antibodies (dark gray box), except for protocol 1, in which tetramer staining was performed in fixed and permeabilized cells together with cytokine staining. In protocol 6, tetramer staining was performed for 2 h at 37°C.

Six different protocols for detecting intracellular cytokines within activated epitope-specific CD4^+^ T splenocytes that differently combined cellular restimulation, tetramer staining, and cytokine labeling were assessed (Figure 1). Protocols were tested in two different experimental settings, in which mice were immunized with H56 or OVA antigens, and the CD4 T-cell response was analyzed employing two different tetramers specific for the H56 and OVA epitopes, respectively.

#### Protocol 1

Splenocytes (2 × 10^6^/well) were cultured in a round-bottom 96-well plate with H56 protein (2 μg/ml) or OVA (50 μg/ml), anti-CD28 and anti-CD49d costimuli (both 2 μg/ml, eBioscience) at 37°C, 5% CO_2_ for 6 h, with brefeldin A (BFA, 5 μg/ml, Sigma-Aldrich) and monensin solution (1 ×, eBioscience) added during the last 5 h of incubation. Following centrifugation at 300 g at 4°C for 7 min, cells were, labeled with Fixable Viability Stain 780 (FVS780, BD Biosciences, 1:1,000, 100 μl/well) for 20 min at room temperature (RT) in the dark, and washed twice in PBS. Cells were fixed and permeabilized for 20 min at 4°C with BD Cytofix/Cytoperm (BD Biosciences). Samples were blocked for 30 min at 4°C in Fc-blocking solution (5 μg/ml of CD16/CD32 mAb, eBioscience, USA) and stained for 1 h at RT with PE-conjugated I-A (b) *M. tuberculosis* Ag85B precursor 280-294 (FQDAYNAAGGHNAVF) tetramer (diluted 1:80, hereafter Tet-Ag85B) or with PE-conjugated I-A (b) chicken ova 325–335 (QAVHAAHAEIN) tetramer (diluted 1:50, hereafter Tet-OVA; both tetramers were kindly provided by NIH MHC Tetramer Core Facility, Emory University, Atlanta, GA, USA) diluted in Perm/wash buffer. In the last 20 min of tetramer incubation, the following mix of fluorescent antibodies was added: APC-conjugated anti-CD3 (clone 145-2C11), BB700-conjugated anti-CD4 (clone RM-5), APC-R700-conjugated, anti-CD44 (clone IM-7), BV786-conjugated anti-IFN-γ (clone XMG1.2), BV650-conjugated anti-TNF-α (clone MP6-XT22), BV421-conjugated anti-IL-17A (clone TC11-18H10), PE-CF594-conjugated anti-IL-2 (clone JES6-5H4) (all antibodies were purchased from BD Biosciences). All antibodies and tetramers were titrated for optimal dilution.

#### Protocol 2

Splenocytes were cultured with the respective antigens and costimuli as in protocol 1 and then washed and stained with the respective tetramers for 1 h at RT. Cells were labeled with FVS780, fixed and permeabilized with BD Cytofix/Cytoperm, and stained with the mix of fluorescent antibodies for 20 min at RT.

#### Protocol 3

Splenocytes were stained with the respective tetramers for 1 h at RT, washed, and stimulated with the respective antigens and anti-CD28 and anti-CD49d costimuli at 37°C for 6 h, with BFA and monensin solution added during the last 5 h of incubation. Cells were labeled with FVS780, fixed and permeabilized with BD Cytofix/Cytoperm, and stained with the mix of fluorescent antibodies for 20 min at RT.

#### Protocol 4

Splenocytes were cultured with the respective tetramers and costimuli for 1 h at RT, washed, and added with BFA and monensin solution at 37°C for 5 h. Cells were labeled with FVS780, fixed and permeabilized with BD Cytofix/Cytoperm, and stained with the mix of fluorescent antibodies for 20 min at RT.

#### Protocol 5

Splenocytes were cultured with the respective tetramers and costimuli for 1 h at RT and for 5 h at 37°C in the presence of BFA and monensin. Cells were labeled with FVS780, fixed and permeabilized with BD Cytofix/Cytoperm, and stained with the mix of fluorescent antibodies for 20 min at RT.

#### Protocol 6

Splenocytes were cultured with the respective tetramers and costimuli for 6 h at 37°C, with BFA and monensin during the last 4 h of incubation. Cells were labeled with FVS780, fixed and permeabilized with BD Cytofix/Cytoperm, and stained with the mix of fluorescent antibodies for 20 min at RT.

### Flow Cytometry

About 7 × 10^5^ stained cells from each protocol were acquired on BD LSRFortessa™ X20 flow cytometer (BD Biosciences) and stored. Data analysis was performed using FlowJo v10 (TreeStar, USA), and the evaluation of different cytokines coexpression was performed using the FlowJo Boolean gate platform. Fluorescence minus one (FMO) controls were performed for all fluorescence and used for gating setting.

### Statistical Analysis

Kruskal-Wallis test, followed by Dunn's post-test for multiple comparisons, was used to assess the statistical difference between protocols. A *P*-value ≤ 0.05 was considered significant. Analyses were performed using GraphPad Prism v7 (GraphPad Software, USA).

## Results

To optimize the protocol for the detection of intracellular cytokines within activated epitope-specific CD4^+^ T cells, we tested different procedures in splenocytes from mice parenterally immunized with two different antigens, the chimeric TB vaccine antigen H56 or OVA, combined with the liposome adjuvant CAF01, 5 days after the booster immunization. The induction of Ag-specific CD4^+^ T cells producing cytokines was assessed, combining antigen restimulation and tetramer staining, followed by intracellular cytokine detection (Figure 1). In protocols 1–3, splenocytes were restimulated with the respective antigens, added before (protocols 1 and 2) or after (protocol 3) tetramer staining. In protocols 4–6, the restimulation step was performed directly with epitope-complexed MHC II tetramers that were therefore used not only as a staining tool but also as a functional stimulus. The comparison of results obtained following the different strategies, and tested with two different antigens, has permitted to optimize the procedure for identifying the cytokine profile of tetramer-specific CD4^+^ T cells.

### Identification of Tetramer-Specific CD4^+^ T Cells Producing Cytokines

**Figure 2:**
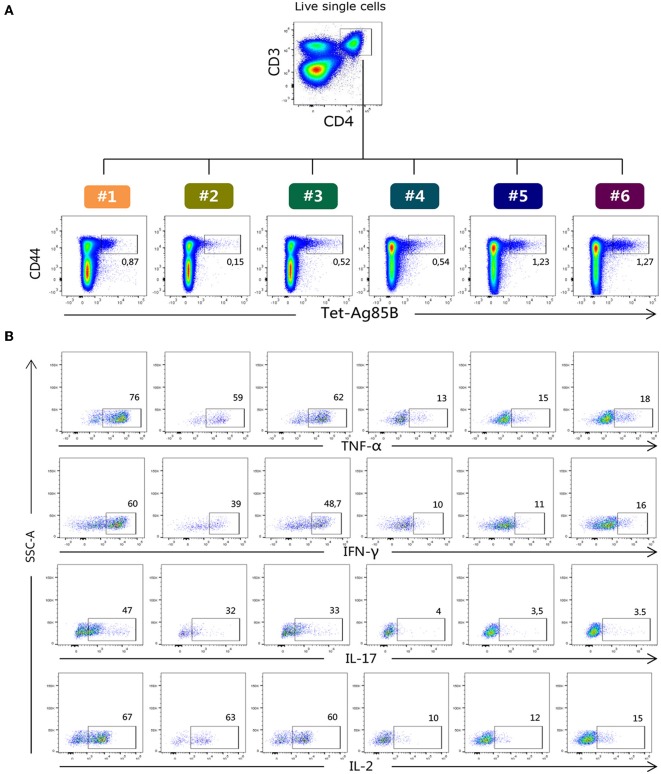
Flow cytometric analysis of Tet-Ag85B^+^ T cells producing cytokines. **(A)** Ag85B-tetramer binding T cells were identified among live single CD3^+^ CD4^+^ as CD44^high^ Tet-Ag85B^+^ cells in the six different protocols, and the frequencies of positive cells are reported within the dot plots. **(B)** Intracellular production of TNF-α, IFN-γ, IL-17, and IL-2 cytokines assessed within the Tet-Ag85B^+^ T cells in the six different protocols. Frequencies of positive cells are reported within the dot plots. Gates were defined on the respective fluorescence minus one (FMO) controls.

H56-specific CD4^+^ T cells were identified using the Ag85B_280−294_-complexed MHC class II tetramers specific for the immunodominant epitope of Ag85B (26), which is part of the chimeric H56 protein, while OVA-specific CD4^+^ T cells were identified using the chicken OVA_325−335_-complexed MHC class II tetramers. Tetramer-positive cells (Tet-Ag85B^+^ or Tet-OVA^+^) were identified as live single CD3^+^ CD4^+^ CD44^+^ cells. All gates were defined on the bases of the respective FMO controls. Staining specificity was determined using a control tetramer complexed with an unrelated antigen that showed a level of staining below 0.02% (data not shown). The identification of Tet-Ag85B^+^ cells in the different six protocols and their intracellular production of TNF-α, IFN-γ, IL-17 and IL-2 are shown in Figures 2A,B respectively.

**Figure 3:**
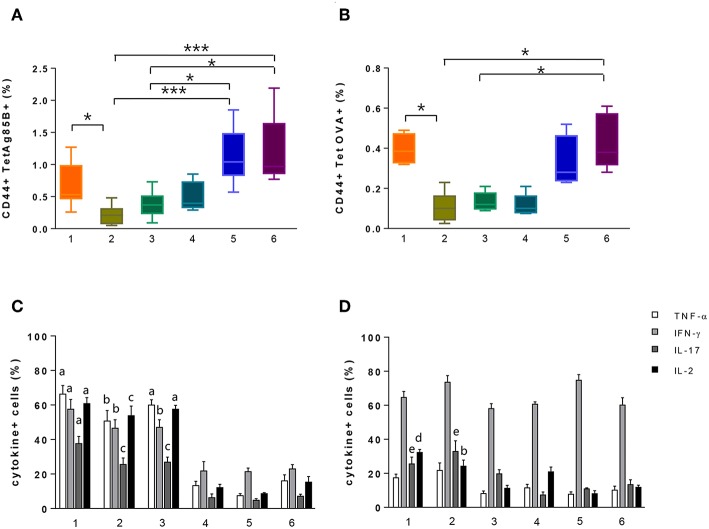
Identification of tetramer-specific CD4^+^ T cells and their cytokine production. Tetramer-specific CD4^+^ T cells and their cytokine production were assessed in splenocytes treated with the different protocols reported in Figure 1. **(A,B)** Box plots of the frequencies of Tet-Ag85B^+^ **(A)** and Tet-OVA^+^ **(B)** T cells with respect to CD4^+^ T cells, detected employing protocols 1–6, as reported in the x axis. Values are reported as mean ± SEM of 10–12 mice, obtained in three independent experiments. Kruskal-Wallis test, followed by Dunn's post-test for multiple comparisons, was used to assess the statistical difference between protocols (*P ≤ 0.05, ***P ≤ 0.001). **(C,D)** Frequencies of TNF-α-, IFN-γ-, IL-17-, and IL-2-positive cells among Tet-Ag85B^+^ **(C)** and Tet-OVA^+^ **(D)** cells, employing protocols 1–6, as reported in the x axis. Values are reported as mean ± SEM of 10–12 mice, obtained in three independent experiments. The significant difference between each cytokine among the different protocols, according to the Kruskal-Wallis test, followed by Dunn's posttest for multiple comparisons (*P* ≤ 0.05), is reported with letters above the error bars; “a” significant difference vs. protocols 4, 5, and 6; “b” vs. protocol 5; “c” vs. protocols 4 and 5; “d” vs. protocols 3, 5, and 6; “e” vs. protocol 4.

ICS protocol typically includes an antigen stimulation step that is crucial for the activation of the effector function of CD4^+^ T cells. Nevertheless, this step induces the internalization of TCR molecules, thus negatively impacting on the tetramer staining procedure. To overcome this limitation, we assessed a strategy based on the antigen stimulation phase followed by permeabilization and fixation of cells and subsequent tetramer staining (Figure 1, protocol 1). Using this procedure that allows the identification of both extracellular and intracellular TCR molecules, we detected 0.53% of Tet-Ag85B^+^ cells (Figure 3A, orange box). This frequency was significantly higher compared to protocol 2, in which splenocytes were first stimulated with H56 antigen and then labeled with the specific tetramer (Figure 1, protocol 2), allowing identification of only 0.2% of tetramer-positive T cells (Figure 3A, light green).

The impact of the tetramer staining performed before antigen restimulation was also evaluated (Figure 1, protocol 3). This procedure allowed detection of a frequency of 0.37% of Tet-Ag85B^+^ T cells that was higher compared to protocol 2 while it was lower with respect to protocol 1 (Figure 3A, dark green box). The higher number of Tet-Ag85B^+^ T cells detected in protocol 1 could be due to the effect of prior antigen restimulation that is known to induce the formation of large clusters of TCR molecules, thus increasing tetramer binding avidity (27).

In protocols 4, 5, and 6, there was no antigen stimulation, and the Ag85B_280−294_-complexed MHC class II tetramers were used not only for identifying but also for stimulating antigen-specific CD4^+^ T cells (Figure 1, protocols 4, 5, and 6). As expected, the frequencies of Tet-Ag85B^+^ T cells detected in protocol 4 were comparable to those of protocol 3 (Figure 3A, light blue and dark green box). In protocols 5 and 6, in which a tetramer incubation phase of 6 h was performed without antigen stimulation, significantly higher frequencies of 1.07 and 1% of Tet-Ag85B^+^ T cells were observed, respect to H56-stimulated samples (Figure 3A, *P* ≤ 0.05 and *P* ≤ 0.001 compared to protocols 3 and 2, respectively). A similar behavior was observed in mice immunized with the OVA antigen, in which the OVA-specific CD4^+^ T cells were identified using the OVA_325−335_ peptide-complexed tetramer (Figure 3B). This shows that tetramers with different peptide specificity respond in a very similar way to the *in vitro* staining procedures assessed in the different protocols.

The effector function of both Tet-Ag85B^+^ and Tet-OVA^+^ T cells identified with the different strategies was analyzed by measuring the intracellular production of four different cytokines using multiparametric flow cytometry. As shown in Figures 3C,D, the highest percentage of cells producing intracellular cytokines was detected for both tetramers in protocols 1 and 2 and for Tet-Ag85B^+^ also in protocol 3 compared to protocols 4, 5, and 6. These data show that in the absence of antigen stimulation, despite the high frequencies of tetramer-binding CD4^+^ T cells (protocols 5 and 6, Figures 3A,B), a significantly lower cytokine production is induced (Figures 3C,D), highlighting the importance of the *in vitro* restimulation step with the vaccine antigen to efficiently stimulate the effector function of antigen-specific CD4^+^ T cells.

### Evaluation of Tetramer-Specific CD4^+^ T Cells Multifunctional Profile

**Figure 4:**
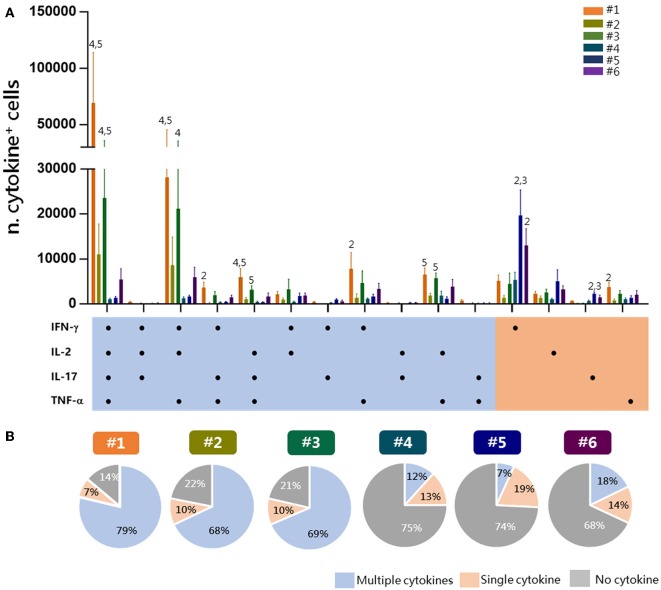
Multifunctional response of Tet-Ag85B^+^ T cells. Multifunctional profiles of Tet-Ag85B^+^ T cells were detected by the different experimental procedures. **(A)** Histograms represent the number of Tet-Ag85B^+^ T cells producing different combinations of cytokines shown on the x axis, detected employing the different protocols. Responses are grouped and color coded according to the functionality (orange for single cytokine, light blue for two or more cytokines). Values are reported as mean ± SEM of 10–12 mice, obtained in three independent experiments, and the numbers above the error bars indicate which protocols are significantly different according to the Kruskal-Wallis test, followed by Dunn's posttest for multiple comparisons (*P* ≤ 0.05). **(B)** Pie charts of the six protocols, in which each slice of the pie represents the fraction of Tet-Ag85B^+^ T cells producing two or more cytokines (multiple cytokines, light blue), a single one (orange), or none (gray). Frequencies are reported within each slice.

To have a picture of the multifunctional profiles of T cells elicited by immunization and detected by the different experimental procedures, a Boolean analysis of data was performed within Tet-Ag85B^+^ cells (Figure 4A). A significant amount of cells positive for all of the four cytokines or for TNF-α, IFN-γ, and IL-2 was observed in protocols 1 and 3 compared to protocols 4 and 5 (*P* ≤ 0.05). Cells producing only IFN-γ were instead significantly higher in protocols 5 and 6 compared to protocol 2 (*P* ≤ 0.01). The analysis of the frequency of cells producing two or more cytokines (multifunctional), a single one (single), or no cytokines in each protocol shows that the frequency of multifunctional Tet-Ag85B^+^ T cells was higher in protocols 1, 2, and 3 (79, 68, 69%, respectively) that included the antigen restimulation. Lower frequencies of multifunctional cells were observed in protocols 4, 5, and 6 (12, 7, 18%, respectively), in which most of the cells did not produce any cytokine (75, 74, 68%) (Figure 4B). The intracellular staining with the tetramer performed in protocol 1 allowed the detection of the highest percentage of multifunctionality, while no differences were observed among protocols 2 and 3. Therefore, the optimal strategy of staining that allows identification of multifunctional T-helper cells among tetramer-specific CD4^+^ T cells was protocol 1.

**Figure 5:**
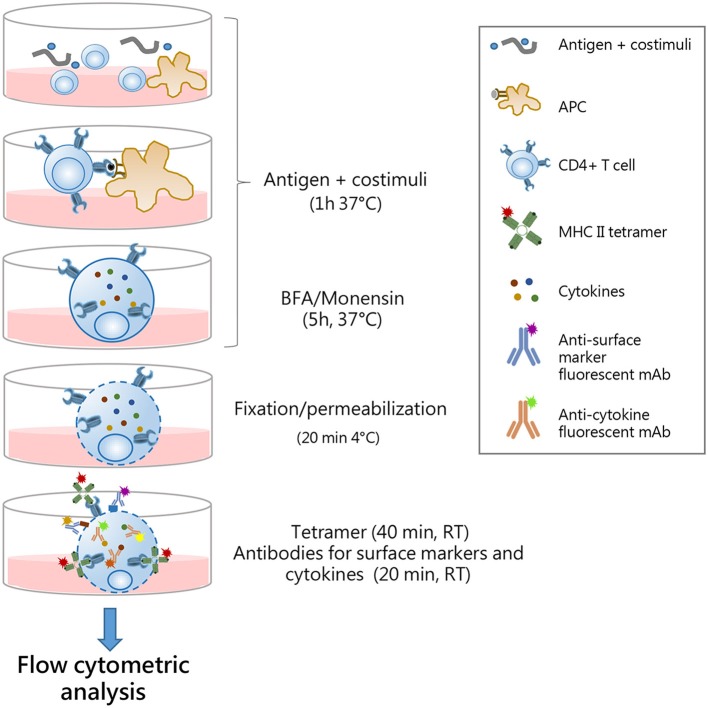
Optimal procedure for identification multifunctional tetramer-specific CD4^+^ T cells. Schematic representation of the protocol optimized for the detection of multifunctional epitope-specific CD4^+^ T cells. Splenocytes are cultured in 96-well plates with antigen and costimuli for 1 h at 37°C to allow antigen presentation by APC to cognate epitope-specific CD4^+^ T cells. Antigen stimulation elicits reactivation of effector function of CD4^+^ cells and TCR internalization. Brefeldin A (BFA) and monensin solution are added for the last 5 h of incubation to block cytokine secretion. Cells are fixed and permabilized for 20 min at 4°C and then simultaneously stained with MHC II tetramers (1 h, at RT) and surface markers/cytokine-specific antibodies (last 20 min). This allows the detection of both surface-expressed and internalized TCR molecules with intracellular cytokines. The single-step staining procedure allows the reduction of the complex time of the protocol. Stained samples are then analyzed by flow cytometry.

In conclusion, our comparative analysis, conducted with two different antigens and their respective tetramers, has shown that the optimal strategy for identifying the multifunctional cytokine profile of tetramer-specific CD4^+^ T cells is the procedure 1, in which the antigen restimulation phase is followed by the intracellular tetramer staining. Indeed, this protocol allows detection of a significant amount of tetramer-specific T cells and their multifunctional activity, and it allows the reduction of the staining time by adding cytokine-specific antibodies in the last 20 min of tetramer incubation. A complete description of protocol 1 is summarized in Figure 5.

## Discussion

In this study, we optimized a flow cytometric protocol to identify at the single-cell level multifunctional epitope-specific CD4^+^ T cells elicited by immunization. Demonstrating pros and cons of different protocols, we showed that the optimal procedure for the simultaneous detection of epitope-specific CD4^+^ T cells and their effector function is based on the antigenic stimulation of cells combined with a single step of cytokine and tetramer staining in permeabilized cells (Figure 5). Our analysis was based on the comparison of different experimental procedures, tested with two different epitope-specific tetramers, in which the steps of antigen restimulation, tetramer and cytokine staining were differently combined. The systematic analysis of different procedures performed in the same samples has offered the possibility of selecting the optimal protocol among different strategies. The results have been confirmed using tetramers specific for two different antigens, thus strengthening the possible application of the selected procedure to the characterization of the complex functional profile of CD4^+^ T-cell responses upon vaccination or infection.

Most of the studies of intracellular cytokine production within tetramer-positive cells have been conducted in CD8^+^ T cells (28–30), while few works have been performed in CD4^+^ T cells with MHC class II tetramers (31–33), and none has compared different protocols in a systematic way. We can generally observe that, in these studies, the tetramer staining was performed extracellularly, often before the antigen stimulation step, even though a direct comparison with the present study is difficult due to different experimental settings, that is, the use of T-cell clones or human CD4^+^ T cells, prolonged incubation with antigen for cell activation, and magnetic bead enrichment of tetramer-positive cells before ICS staining. Our analysis clearly demonstrates that antigenic stimulation is necessary for an efficient reactivation of the cellular effector function, and the same stimulation effect cannot be obtained with the direct incubation of cells with epitope-complexed MHC tetramers, also when prolonged for 6 h (protocols 5–6). Nevertheless, many studies have demonstrated that ligation of TCR by processed antigen induces TCR internalization and a subsequent downmodulation of its cell surface expression (19, 34). Indeed, in protocol 2, in which antigen stimulation was performed before the extracellular tetramer staining, the frequency of tetramer-positive cells was significantly lower compared to that in protocol 1.

Here, using tetramers specific for two different antigens, we have shown the efficiency of tetramer staining performed in permeabilized cells (protocol 1) that allows detection of both surface-expressed and internalized TCR molecules, resulting in the identification of the highest percentages of tetramer-binding cells. This procedure stained epitope-specific CD4^+^ T better than protocol 3 in which labeling with tetramer was performed before antigen stimulation. This can be due to the lower avidity of tetramer binding to TCR molecules in the absence of cell activation by antigen stimulation. Indeed, cellular activation is known to induce the TCR reorganization with the generation of large clusters of TCR molecules (27) that increase the strength of tetramer binding.

Analysis of multifunctional CD4^+^ T cells is of critical importance for in-depth characterization of immune responses to vaccination both in preclinical and clinical studies. It is therefore essential to have a protocol that optimally combines the identification of antigen-specific T cells with the analysis of their cytokine profile. Here, we show the possibility to combine, upon antigen stimulation, tetramer, and intracellular cytokine staining in permeabilized cells, allowing the identification of a higher number of polyfunctional tetramer-positive CD4^+^ T cells. The amount of cells producing all of the four cytokines or coexpressing two or three cytokines (especially TNF-α, IFN-γ, and IL-2) was indeed higher compared to the other protocols tested. Significantly lower levels of multifunctional cells were observed when tetramers were used both as stimulus and as staining (protocols 4, 5, and 6). Indeed, even though a higher percentage of tetramer-binding T cells was identified by protocols 5 and 6, about 70% were negative for cytokine production, with respect to 14% observed in protocol 1, demonstrating that the binding of epitope-complexed MHC class II molecules to TCR in the presence of CD49d and CD28 costimuli is not sufficient to effectively reactivate multifunctional antigen-specific CD4^+^ T cells.

The functional characterization of CD4^+^ T cells described by the protocols analyzed here is particularly suitable for preclinical studies in which sufficient quantity of CD4^+^ T cells can be easily identified in draining lymphoid organs such as lymph nodes or spleens, while in humans, it is generally more complicated because the frequency of antigen-specific CD4^+^ T cells in blood is low and often undetectable (6). Moreover, the use of MHC tetramers requires prior knowledge of the peptide epitope and host MHC haplotype, a limitation that can be easily circumvented in inbred animals.

In conclusion, in the present work, we have selected an optimized protocol for identifying epitope-specific CD4^+^ T cells and their effector function, combining antigenic stimulation of cells with the intracellular staining of TCR molecules and cytokines. Antigenic restimulation, performed at the beginning of the procedure, allows the activation of cells and elicits multiple cytokine production, but at the same time, it promotes the downregulation of surface TCR expression that is resolved by the intracellular tetramer staining. This procedure allows also reduction of the total protocol time since tetramer, surface marker, and cytokine staining are combined in a single staining step.

This protocol allows better understanding of the complex functional profile of T-cell responses upon vaccination or natural infection, and it can be instrumental for the dissection of the immune response to vaccination.

## Data Availability Statement

The datasets generated for this study are available on request to the corresponding author.

## Ethics Statement

This study was carried out in accordance with the recommendations of Italian Ministry of Health (authorization n° 1004/2015-PR, 22 September 2015).

## Author Contributions

GP, DM, and AC conceived and designed the experiments. GP, MC, and EP performed the experiments. GP, MC, AC, EP, and EN analyzed the data. GP and AC wrote the paper. DM and AC critically revised the manuscript. All authors read and approved the final manuscript.

### Conflict of Interest

The authors declare that the research was conducted in the absence of any commercial or financial relationships that could be construed as a potential conflict of interest.
